# Evaluation of Piezoresistive and Electrical Properties of Conductive Nanocomposite Based on Castor-Oil Polyurethane Filled with MWCNT and Carbon Black

**DOI:** 10.3390/ma16083223

**Published:** 2023-04-19

**Authors:** Diego S. Melo, Idalci C. Reis, Júlio C. Queiroz, Cicero R. Cena, Bacus O. Nahime, José A. Malmonge, Michael J. Silva

**Affiliations:** 1 Department of Physics and Chemistry, Faculty of Engineering, São Paulo State University (UNESP), Ilha Solteira 15385-000, SP, Brazil; 2 Department of Energy Engineering, Faculty of Engineering and Science, São Paulo State University (UNESP), Rosana 19274-000, SP, Brazil; 3 Science and Technology Goiano, Federal Institute of Education, Rio Verde 75901-970, GO, Brazil; 4 Institute of Physics, Federal University of Federal do Mato Grosso do Sul (UFMS), Campo Grande 79070-900, MS, Brazil

**Keywords:** conductive nanocomposite, castor-oil polyurethane, multiwall carbon nanotube, carbon black, piezoresistive sensor

## Abstract

Flexible films of a conductive polymer nanocomposite-based castor oil polyurethane (PUR), filled with different concentrations of carbon black (CB) nanoparticles or multiwall carbon nanotubes (MWCNTs), were obtained by a casting method. The piezoresistive, electrical, and dielectric properties of the PUR/MWCNT and PUR/CB composites were compared. The dc electrical conductivity of both PUR/MWCNT and PUR/CB nanocomposites exhibited strong dependences on the concentration of conducting nanofillers. Their percolation thresholds were 1.56 and 1.5 mass%, respectively. Above the threshold percolation level, the electrical conductivity value increased from 1.65 × 10^−12^ for the matrix PUR to 2.3 × 10^−3^ and 1.24 × 10^−5^ S/m for PUR/MWCNT and PUR/CB samples, respectively. Due to the better CB dispersion in the PUR matrix, the PUR/CB nanocomposite exhibited a lower percolation threshold value, corroborated by scanning electron microscopy images. The real part of the alternating conductivity of the nanocomposites was in accordance with Jonscher’s law, indicating that conduction occurred by hopping between states in the conducting nanofillers. The piezoresistive properties were investigated under tensile cycles. The nanocomposites exhibited piezoresistive responses and, thus, could be used as piezoresistive sensors.

## 1. Introduction

Owing to the increasing environmental concerns and decreasing fossil fuel sources, the scientific community and industry search for sustainable sources to produce new materials. Materials obtained from renewable and sustainable sources, such as bio-based and natural polymers, have attracted considerable interest in recent decades. These include natural rubber [1], cellulose [2], polypropylene based on cane alcohol [3], and polyurethane based on castor oil (PUR) [4].

Among bio-based polymers, PUR attracts considerable attention because it uses vegetable oils as polyols obtained from natural sources [5]. Castor oil is attractive because it has a high quantity of hydroxyl groups (–OH) that react with isocyanate (–NCO), forming urethane bonds [6]. These hydroxyl groups are derived from ricinoleic acid, which constitutes approximately 80% of the oil. The hydrophobic nature of the triglycerides in the castor oil contributes to its excellent mechanical properties, including good elongation and tensile strength [6,7,8]. Owing to its good characteristics, a vegetable-oil-based PUR has been used as a polymeric matrix to obtain composite materials for different applications, such as adhesive resins [9,10], coatings [11,12], electromagnetic interference shielding materials [13], materials for sorption of oils and organic solvents [14], and conductive polymer composites (CPCs) [15,16].

A conventional polymer is combined with a conductive filler to form CPC, combining good mechanical properties with an easier processing of polymeric matrix and the excellent electrical properties of the conducting fillers [17]. Nonetheless, the electrical conductivity of CPCs is influenced by many factors, such as the preparation method (quantity and distribution of conductive fillers in the host matrix), as well as the volume fraction and electrical conductivity of the phase [18,19]. For instance, when the conductive phase concentration is extremely small, a considerable distance exists between each conducting filler, which results in a composite with a similar electrical conductivity as a polymer matrix [20,21]. However, if the concentration of conducting fillers is high, an insulator-to-conductor transition will occur, resulting in the formation of a continuous conducting path. It is at this point that percolation threshold can be observed, and the composite’s electrical conductivity is approximately equal to the conductive fillers [21,22]. There are two main mechanisms that determine electrical conduction in CPCs: the percolation model for tunneling and hopping conduction [23]. The model of hopping conduction describes the charge carriers jumping between localized states in the conducting particles or aggregates in contact with each other forming a three-dimensional structure in the bulk of the composite [24]. In tunneling conduction, conductive particles and/or aggregates are separated by insulating layers or barrier potentials [25].

In this sense, for the production of a CPC-based PUR, several conductive fillers can be used, including carbon-based nanomaterials such as expandable graphite [26], multi-wall carbon nanotubes (MWCNTs) [27,28], graphene oxide [29,30,31], and carbon black (CB) [16,32]. For example, Dai et al., 2020 obtained a MWCNT/PUR nanocomposite using a solvent-free method and showed that, owing to the interaction between the polymer matrix and MWCNT, the tensile strength and thermal stability were improved for MWCNT loadings up to 0.5 wt%. Kumar et al., 2022 evaluated the ameliorating properties of PUR-based nanocomposites via a synergistic addition of graphene and cellulose nanofibers. Consequently, these nanocomposites can be applied to protective coatings and automobile parts [33]. Min et al., 2023 conducted a study in which a highly electrically conductive composite film was fabricated by shearing the MWCNT/PDMS paste in two rolls. The authors report that the electrical conductivity and activation energy of the composite were 326.5 S/m and 8.0 meV, respectively, at 5.82 vol% of MWCNTs [34]. Another study examined how structural factors affected the electrochemical performance of carbon composites [35]. Carbonization of binary composites formed by graphene nanoplatelets and melamine (GNP/MM), multi-walled carbon nanotubes and melamine (CNT/MM), and trinary composites (GNP/CNT/MM) plays a crucial role in tailoring electrochemical properties of carbon hybrid materials, which are considered noble metal-free alternatives to traditional electrodes [36]. In a study conducted by Rozhin et al., 2023, single-walled carbon nanotubes (SWCNTs) were compared with double-walled carbon nanotubes (DWCNTs) as nanostructured additives for tripeptide hydrogels. It has been found that carbon nanomaterials (CNMs) can enhance the viscoelastic properties of tripeptide hydrogels, but their presence can also impair the self-assembling process [36].

Due to the excellent mechanical properties, lightness, and flexibility of polymer matrices and the good electrical properties of carbon-based nanofillers, composite materials obtained through this combination have the potential to be used as piezoresistive sensors. Piezoresistivity can be defined as an electromechanical phenomenon in which the electrical resistance of a material changes reversibly under a strain cycle [37]. In this sense, the piezoresistivity consists of a change in the conductive composite structure with strain, and it is a material property associated with a change in the structure and resistivity of material [37]. Changes in the degree of electrical continuity in the conductive composite are commonly associated with reversible microstructural changes. Many applications require the detection of strain or stress on structures, such as structural vibration control, traffic monitoring, weighing (including weighing in motion), and building facility management [37].

Several studies used PUR as a matrix and second-phase carbon-based nanofiller, while few studies investigated the electrical, dielectric, and piezoresistive properties of this type of nanocomposite. Using two different carbon-based nanofillers (CB and MWCNT), this project aims to obtain a castor oil-based polyurethane nanocomposite for use as a piezoresistive sensor. A simple and low-cost synthesis route was used to obtain the specimens through the casting method. A comparative analysis was conducted between nanocomposites with different concentrations of conductive nanofiller in order to determine which nanocomposites provide the best results in electrical, dielectric, and piezoresistive measurements. Additionally, the results showed that thin, flexible films with low percolation thresholds were obtained for the PUR/MWCNT and the PUR/CB nanocomposites, making them suitable for applications as sensors, anti-static (electrically conducting), shape memory alloys, and electromagnetic-wave shielding.

## 2. Materials and Methods

### 2.1. Materials

A bicomponent castor-oil-based polyurethane was purchased from Sinergia LTDA, city of Araraquara, SP, Brazil. A mixture of prepolymer (component A) and polyol (component B) was used to obtain a pure PUR film with a mass fraction B/A of 2/1.

Conducting CBs (Printex XE-2) were purchased from Degussa (Paulínia, Brazil). They had a surface area of 1000 m^2^/g, average particle size of 35 nm, and bulk density of 0.1 g/cm^3^. MWCNTs were supplied by CTNANO, Federal University of Minas Gerais (UFMG) in the city of Belo Horizonte, MG, Brazil. They had a surface area of 100 m^2^/g, bulk density of 0.23 g/cm^3^, and average diameter and length of 20 nm and 5–30 µm, respectively. Before use, both conductive fillers were milled.

### 2.2. Conducting Nanocomposite Preparation

A neat PUR was prepared by mixing components A and B in a mass proportion of 1 g/2 g (isocyanate component/polyol) in 1 mL of chloroform (Dynamics Analytical Reagents). PUR/CB and PUR/MWCNT nanocomposite samples were prepared at a constant PUR mass fraction (3 g) while varying the mass concentrations of MWCNT and CB nanoparticles. CB and MWCNT values used in the sample preparation can be found in Table 1.

**Figure 1:**
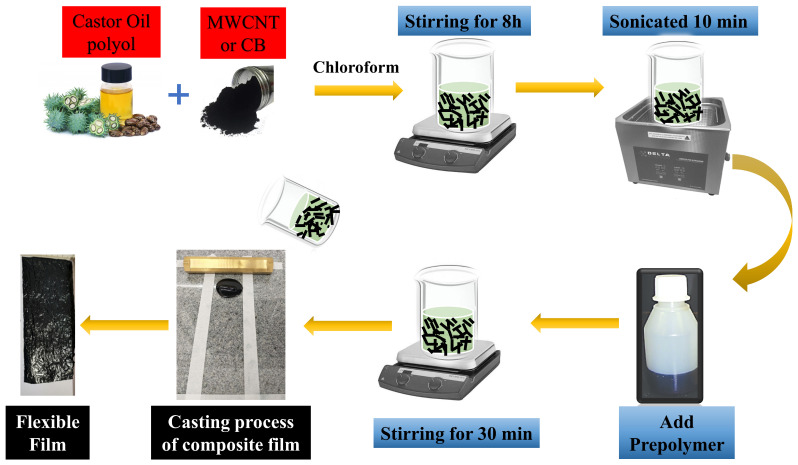
Schematic representation of the preparation of conducting nanocomposite samples.

Figure 1 illustrates the preparation diagram for nanocomposites specimens. In 1.0 mL of chloroform, polyol (2 g) was mixed with different concentrations (1–5 mass%) of the conductive filler (MWCNT or CB). After 8 h of stirring, the prepolymer was added to the polyol/conductive filler/chloroform dispersion and stirred for 10 min before it was sonicated for 10 min. Flexible samples, with thicknesses of 100–200 μm, were obtained by casting on glass slides and curing for 5 days at room temperature.

### 2.3. Characterization

The morphology of PUR/CB and PUR/MWCNT nanocomposite films were analyzed by scanning electron microscopy (SEM) using an EVO LS15 Zeiss microscope. SEM analysis was performed after cryo-fracturing samples with liquid nitrogen and drying them under dynamic vacuum. Samples were coated with a thin layer of carbon (10 nm) using the sputtering method.

A voltage/current source from Keithley Instruments model 247 (high-voltage supply) was used to measure direct-current (DC). On both sides of the film, the electrodes were painted with conductive paint for electrical contact. The DC electrical conductivity was calculated by Equation (1):

(1) $$\sigma_{dc}=\frac{I}{V}\frac{d}{A}$$

where *A* is the electrode area, *d* is the sample thickness, and *V* and *I* are the applied voltage and measured current, respectively.

A Solartron SI 1260 impedance analyzer with a 1296 dielectric interface (0.05% basic accuracy) was used to measure the alternating-current (AC) electrical and dielectric properties of the PUR/MWCNT and PUR/CB nanocomposite samples. An impedance analysis of the nanocomposites was performed in a frequency range of 0.1–10^6^ Hz, at a voltage of 1 V, at room temperature.

At room temperature, piezoresistive tests were conducted by measuring the electrical resistances of the PUR/MWCNT and PUR/CB nanocomposite samples, using Keithley 237 high-voltage (0.3% basic accuracy) source-connected copper electrodes, during a uniaxial mechanical deformation of the samples. This test was performed using a universal testing machine from Instron (model 3639, 0.15% and 0.2% basic accuracy of set speed and displacement) in accordance with the International Organization for Standardization (ISO) 37:2011 standard, with a 100-N load cell. The copper electrodes (10 mm × 5 mm × 0.2 mm) were wrapped around both upper and lower ends of the samples and fixed using clamps on the mechanical testing machine. Piezoresistive measurements were performed at an applied voltage of 10 V, deformation of 10%, and at a velocity of 100 mm/min. The samples were cut in accordance with ISO 1286:2006. Piezoresistive tests were carried out on PUR/MWCNT and PUR/CB nanocomposite samples containing 3, 4, and 5 mass% MWCNT and CB.

## 3. Results and Discussion

### 3.1. Morphological Analysis

**Figure 2:**
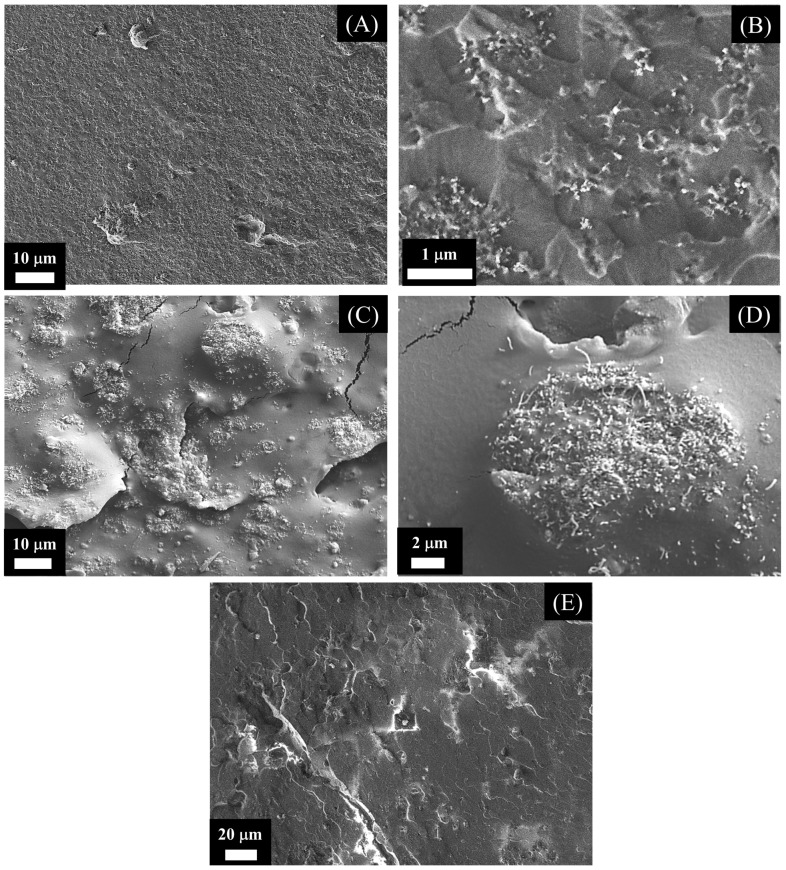
SEM microimages of (**A**,**B**) PUR/CB and (**C**,**D**) PUR/MWCNT nanocomposite samples with 5 mass% of conductive nanofillers and (**E**) Neat PUR.

Figure 2 shows SEM images of cryo-fractured surfaces of PUR/CB and PUR/MWCNT nanocomposite samples with different concentrations of conductive nanofillers. As shown in Figure 2A–D, the MWCNT fillers tended to cluster more than CB. The formation of MWCNT agglomerates has been attributed to van der Waals interactions between the fillers [15,38]. Nayak et al. observed a similar behavior when the CNT concentration was greater than 3% in the polyamide matrix [38]. On the other hand, the CB nanoparticles exhibited a more homogenous dispersion in the PUR matrix. Nevertheless, agglomeration of CB was also observed. Compared to MWCNTs, CB nanoparticles display better dispersion, and aggregates are easier to break due to their weak electrical interaction [39,40].

### 3.2. DC Electrical Conductivity

A CPC can be created by adding conductive particles to an insulating polymeric matrix. Various factors contribute to the electrical properties of composites and the electrical conduction process, including the preparation of CPC, electrical conductivity, and the volumetric fraction of the phase, particle size, and aggregate size, as well as their dispersion within polymeric matrixes [15,41]. Generally, nanoparticles homogeneously dispersed within a matrix produce a CPC with a lower percolation threshold [41].

At extremely low conductive particle concentrations, the distances between the conductive fillers or aggregates are considerable, and the CPC exhibits a similar electrical conductivity to the polymeric host. When the concentration of conductive filler reaches a critical level (or percolation threshold), an insulator–conductor transition occurs, resulting in the formation of a percolation network or continuous conductor path through which charge carriers move when an external electric field is applied.

**Figure 3:**
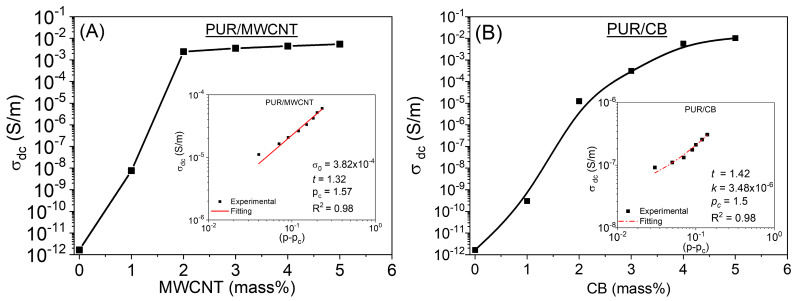
*σ_dc_* behavior in relation to the conductive nanofiller mass fractions for (**A**) PUR/MWCNT and (**B**) PUR/CB nanocomposite. The Figure presents the double-logarithmic plot, based on Equation (2), for the PUR/MWCNT nanocomposite and for the PUR/CB nanocomposite.

Figure 3 illustrates this behavior for both PUR/MWCNT and PUR/CB nanocomposites containing different amounts of conductive nanofillers (MWCNT or CB). PUR/CB nanocomposite samples showed an insulator–metal transition at slightly lower filler concentrations than PUR/MWCNT nanocomposite samples, likely due to the fact that CB tends to cluster less than MWCNTs, as shown in the SEM images. There is a distinct difference between the dc electrical conductivity curve profiles of the two types of nanocomposites. PUR/MWCNT nanocomposite reaches maximum conductivity at around 2 mass% MWCNT in the PUR matrix, while PUR/CB reaches maximum conductivity at around 5 mass%. The reason for this behavior can be attributed to the morphology and dispersion of the nanoparticles within the PUR matrix. In the PUR/CB and PUR/MWCNT nanocomposite samples, the percolation thresholds calculated were 1.56 and 1.50 mass% of MWCNT or CB, respectively. For both types of nanocomposites, the maximum conductivity was approximately 10^−2^ S/m, which is approximately 9-fold greater than the conductivity of PUR.

The *σ_dc_* behavior of PUR/MWCNT and PUR/CB nanocomposites, composed of both insulating and conductive phases, can be described by power-type equations. When the conductive fillers fraction reaches the critical volume fraction pc in the percolation threshold region, geometric connectivity begins to form throughout the system, resulting in uninterrupted and continuous conductive paths [20,42]. Thus, the *σ_dc_* behavior of a nanocomposite can be expressed by:

(2) $$\sigma_{dc}=k{\left(p-p_{c}\right)}^{t}$$

where *p* is the concentration of the conductive phase, *k* is the preexponential constant, and *t* is the critical conductivity exponent. For two-dimensional systems, its value is 1.3–1.5, while, for three-dimensional systems, *t* is 1.6 to 2.0 [43]. Using the data of Figure 3A,B, we generated the graph of log (*σ_dc_*) as a function of log (*p – p_c_*), while the fitting was performed using Equation (2). A linear fit was performed on the points of inside graphics of Figure 3A,B to estimate the *t* and *k* values for the PUR/MWCNT and PUR/CB nanocomposite samples, in which the *t* values were 1.32 and 1.42, while the *k* values were 3.82 × 10^−3^ and 3.48 × 10^−6^, respectively.

The *t* values for the PUR/MWCNT and PUR/CB samples agree with the universal percolation theory [44]. The electrical conduction process of charge carriers occurs through the conductive two-dimensional network by geometric contact between the CB nanoparticles in the PUR/CB nanocomposite samples. Similar behavior occurs for PUR/MWCNT nanocomposite samples in which the conductive two-dimensional network is formed by geometric contact of the MWCNTs [45,46].

Based on analytical micromechanical analysis, Mazaheri et al. predicted the electrical conductivity and the percolation behavior of polymer nanocomposites containing spherical carbon black nanoparticles as fillers. In the proposed model, quantum electron tunneling is accounted for, as well as the thickness of the interphase region, the radius of the filler, the conductivity of the filler, the conductivity of the interphase region, and the conductivity of the matrix [47]. In addition to the geometrical and physical properties of the CB fillers, polymer matrix, and interphase layer, the authors also validated the analytical formulation of the model [47]. The model produces meaningful physical results by accounting for electrical conductivity, potential barrier height, and tunneling distance within the interphase region [47]. The model described the behavior of electrical conductivity in both insulating and percolation transition regions, at very low volume fractions, accurately [47]. In a study by Zare and Rhee, the electrical conductivity of CNT-filled samples was tuned using a mechanics model, assuming extended CNT. A study of the extended CNT was conducted by considering the interphase and tunneling areas and estimating the conductivity of prolonged CNT based on the resistances of conductive nanofiller, interphase, and tunneling districts [48]. In nanocomposite systems, conductivity directly depends on the network size and interphase depth, but electrical conductivity of CNT and interphase are ineffective [48]. In addition, the narrow and undersized tunnels benefit the nanocomposite’s conductivity because of its low percolation onset and little tunneling resistance [48].

### 3.3. Impedance Spectroscopy (IS)

IS is a useful technique for examining the dielectric and electrical properties of various types of materials. IS can be used to investigate the dependence between the electrical properties of a material and frequency of the applied electric field for an extensive range of frequencies (from 10^−4^ to 10^7^ Hz) [49]. This technique is relatively easy to use, and results can be related to polarization effects, dielectric properties, microstructure, defects, and electrical conduction mechanisms [49]. It is possible to determine the electrical and dielectric behaviors of a material from a set of values related to the complex impedance formalism (Z*) expressed by:

(3) $$Z^{*}=Z^{\prime }\;+\;iZ^{\prime \prime }$$

where *Z*′ and *Z*″ are the real and imaginary parts of the complex impedance, respectively, and *i* is the imaginary unit (√−1). The sets of values obtained from Z* are the (a) complex admittance (Y*), (b) complex dielectric permittivity (ε*), (c) complex electrical module (M*), and (d) complex electrical conductivity (σ*) [49]. The Z* formalism is related to each other by the interrelation factor *μ* = *jωC*_0_, where *C*_0_ is the vacuum capacitance, *ω* is the angular frequency, and *j* (√−1) is the imaginary factor.

**Figure 4:**
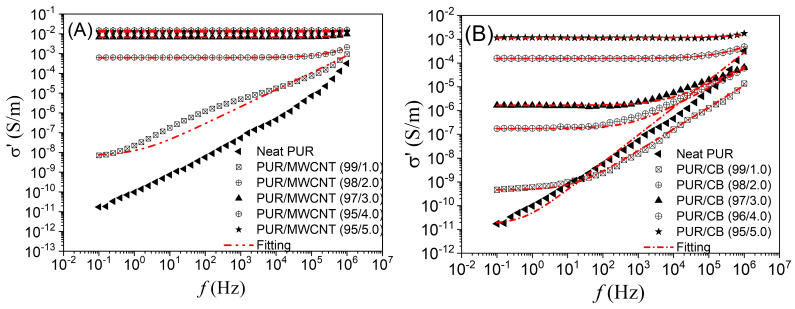
Real part of complex electrical conductivity as a function of frequency for (**A**) PUR/MWCNT and (**B**) PUR/CB with different mass fractions of the conductive nanofiller.

Figure 4 shows the real parts (*σ*′(*f*)) of the complex electrical conductivity with respect to the AC electrical field frequency for PUR/MWCNT and PUR/CB nanocomposites. It should be noted that both nanocomposites exhibit *σ*′(*f*) similar to disordered solids, i.e., frequency-dependent electrical conduction, primarily, at high frequencies [50,51,52]. This behavior has been most evident in samples with nanofiller concentrations below 3 mass%.

As illustrated in Figure 4A,B, the PUR/MWCNT and PUR/CB nanocomposite samples, with different quantities of conductive nanofillers, exhibited two well-defined regions of the *σ*′(*f*) curve, namely a frequency-independent region and a frequency-dependent region. According to Kilbride and Coleman, the frequency-independent behavior of *σ*′(*f*) can be attributed to the variation in the correlation length with the conductive nanofiller content, which has been discussed in relation to a biased random walk in a three-dimensional network [53]. In this case, the frequency-independent *σ*′(*f*) behavior in the low-frequency region reflects a large-distance transport of charge carriers via conducting percolative paths inside the nanocomposite, which is improved by the electric-field concentration effect [54].

For the PUR/MWCNT and PUR/CB samples, a plateau is observed in the *σ*′(*f*) curve at low frequencies, which is approximately equal to *σ_dc_* [1,54]. However, the transition (critical frequency) from the frequency-independent to the frequency-dependent *σ*′(*f*) shifted to a higher frequency with the increase in the concentration of the conductive nanofiller dispersed in the nanocomposite, which may be related to the formation of a conductive two or three-dimensional network with increasing concentrations of both MWCNT and CB nanoparticles.

The frequency-dependent behavior of PUR/MWCNT and PUR/CB nanocomposite samples occurs when the critical frequency is reached. *σ*′(*f*) increases with frequency due to the hopping conduction of charge carriers between more closely located states with lower energy barriers, as well as the effect of spatial charge polarization at the nanofiller–PUR interface [53,55]. For samples containing concentrations above 2 mass% of MWCNT and 3 mass% of CB, the critical frequency (which normally occurs at higher frequencies) could not be obtained due to the conductive nature of the nanocomposites. This is due to limitations of the equipment needed to study at higher frequencies.

The *σ*′(*f*) behavior in Figure 4A,B, for both nanocomposites, can be described by the Jonscher’s power law:

(4) $$\sigma^{\prime }\left(f\right)=\sigma_{dc}+A\omega^{n}$$

where $\sigma_{dc}$ is the DC electrical conductivity (plateau or frequency-independent region), *A* is the preexponential factor, related to the strength of polarizability, and *n* is the fractional exponent that can vary between 0 and 1 [56]. The exponent *n* is related to the degree of interaction between the charge carriers and the networks around them, suggesting that electrical conduction may occur through hopping and/or interfacial polarization [57,58].

Table 2 presents the results of fitting log σ′(*f*) versus log (frequency) curves (Figure 4A,B) using Equation (4) for all nanocomposite samples. According to the results, the *n* values ranged from 0.7 to 1.0, indicating that electrical conduction involves the hopping of charges between localized states or spatial charges trapped at the interface between the PUR matrix and the conductive nanofillers [56,57].

**Figure 5:**
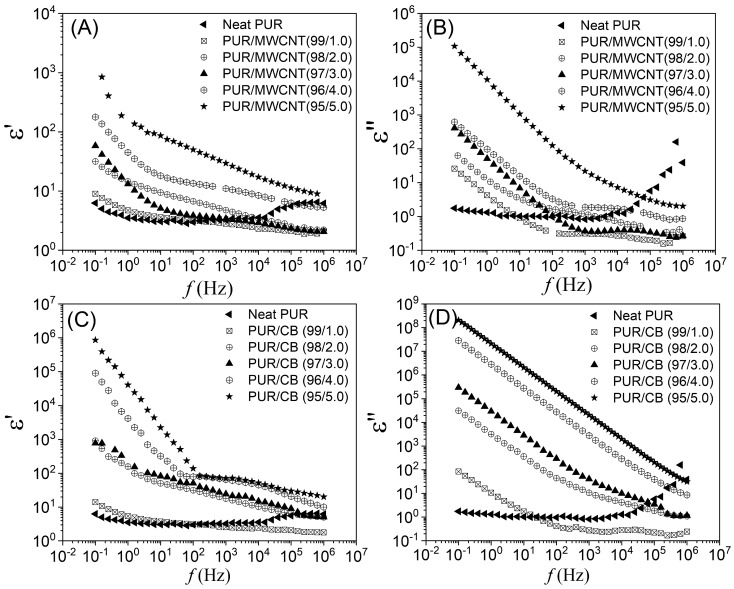
Complex dielectric constant, as a function of frequency for (**A**,**B**) PUR/MWCNT and (**C**,**D**) PUR/CB, with different mass fractions of the conductive nanofiller.

The frequency-dependence of the real ($\varepsilon^{\prime }$) and imaginary ($\varepsilon^{\prime \prime }$) dielectric constants of PUR/MWCNT and PUR/CB nanocomposite samples, measured at room temperature, is shown in Figure 5. The $\varepsilon^{\prime }$ represents the ability of a material to become polarized in the presence of an electric field, whereas the $\varepsilon^{\prime \prime }$ represents the dielectric losses incurred by a material due to its increased conductivity. Figure 5A,C illustrates the behavior of $\varepsilon^{\prime }$ as a function of frequency for PUR/MWCNT and PUR/CB nanocomposites, respectively. At a higher concentration of conductive fillers, more charge carriers are trapped at the conductive filler–polymer matrix interfaces. In this regard, when the CPC is under the influence of electric fields, electrons move to and accumulate at one end of the conductive cluster (filler–matrix interface), while the other end is more positive, which generates a dipole moment across the clusters [59]. On the other hand, when the electric field is overturned, the reverse behavior occurs. However, as the frequency of the electric field increased, the residence time of the trapped electrons at the interface decreased, resulting in a decrease in the polarization of the system [15]. This behavior can be observed as a reduction in $\varepsilon^{\prime }$ with the increase in the frequency, particularly in the high-frequency range, for all nanocomposite samples. This decrease is principally attributed to the Maxwell–Wagner–Sillars (MWS) polarization and space charge polarization in the bulk nanocomposite samples [60,61].

Figure 5B,D shows the frequency dependences of $\varepsilon^{\prime \prime }$ for the PUR/MWCNT and PUR/CB nanocomposite samples, respectively. In the range of low frequencies, a significant increase in $\varepsilon^{\prime \prime }$ can be observed due to the increasing conductivity of the nanocomposite samples [62,63]. The dielectric loss comes from the fact that the charge carriers are no longer participating in the sample polarization process and are starting to participate in the conduction process through the percolative conductive network in the composite [15]. Asandulesa et al., 2021 observed similar behavior for ε’ and ε” as a function of frequency for PTB7:PC71BM photovoltaic polymer blend. According to the authors, a high dielectric constant reduces the Coulomb attraction between electrons and holes in excitons and donor–acceptor exciplexes, thereby increasing the power conversion efficiency (PCE) [64].

In Table 3, the values of $\varepsilon^{\prime }$ and $\varepsilon^{\prime \prime }$ are shown at frequencies of 1 kHz and 1 MHz for both conductive nanocomposites. As seen, the values of $\varepsilon^{\prime }$ and $\varepsilon^{\prime \prime }$ were higher for samples with concentrations higher than the percolation threshold, mainly for samples 96/4.0 and 95/5.0. As a result of (i) having a greater amount of charge carriers trapped in the polymer-nanofiller interface, as well as (ii) the samples becoming more conductive as the nanofiller amount increased, the samples became more conductive. Asandulesa et al., 2020 observed similar behavior for $\varepsilon^{\prime }$ and $\varepsilon^{\prime \prime }$ as a function of frequency for the PTB7:PC71BM photovoltaic polymer blend. According to the authors, a high dielectric constant reduces the Coulomb attraction between electrons and localized states [65].

### 3.4. Piezoresistivity Analysis

**Figure 6:**
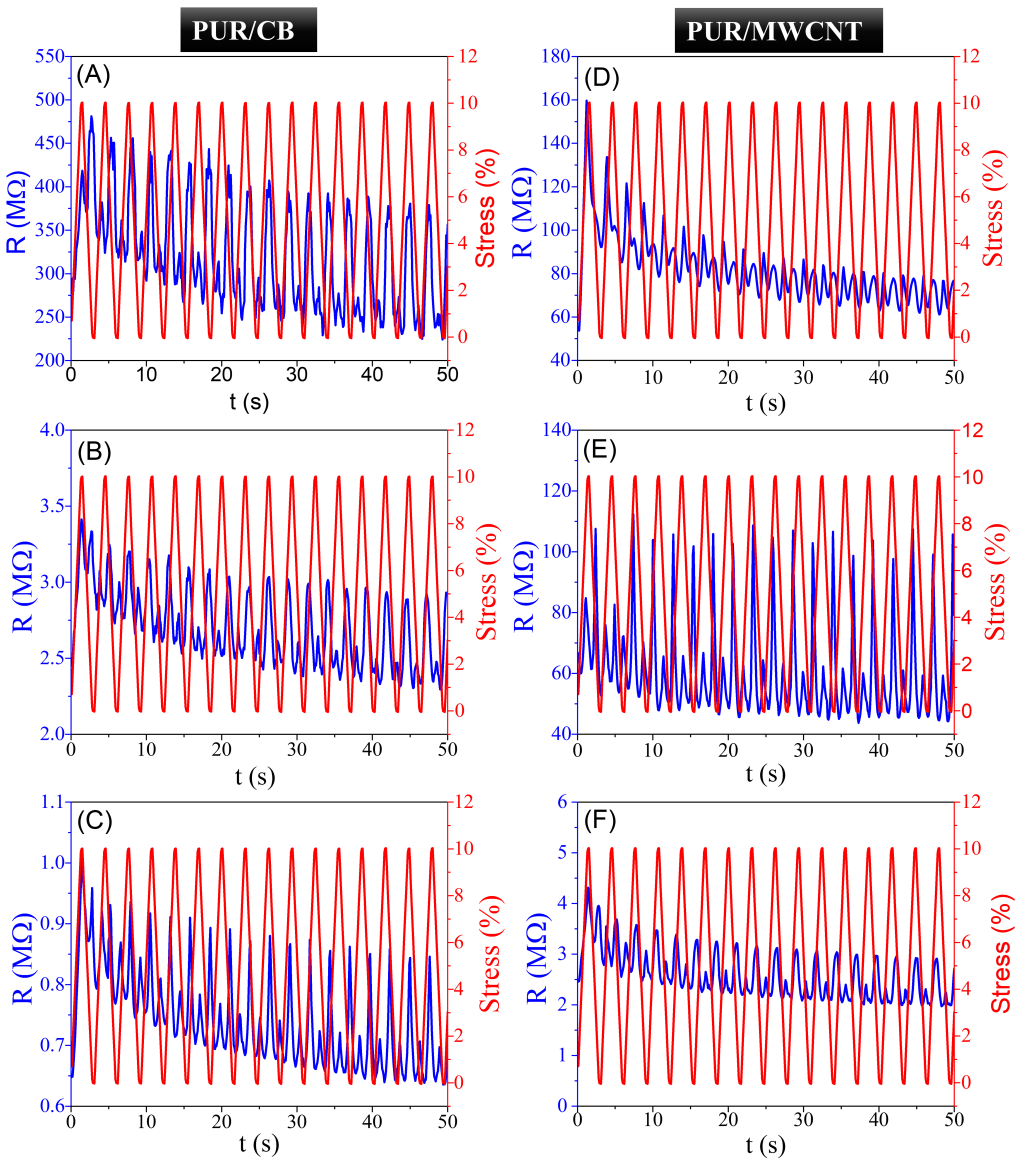
Piezoresistive response of (**A**–**C**) PUR/CB and (**D**–**F**) PUR/MWCNT nanocomposite with 3, 4 and 5 mass% for 10% of strain and 16 cycles.

In-situ piezoresistance measurements were performed while mechanically stretching the PUR/MWCNT and PUR/CB nanocomposite films across the elastic region. In Figure 6, a variation in electrical resistance is observed during the elastic regime for PUR/MWCNT and PUR/CB nanocomposite samples containing 3, 4, and 5 mass% of MWCNT and CB, over 16 cycles of loading and unloading. During each cycle, the deformation of the film increased linearly with the applied strain. It was observed that the slope was similar during unloading, as well, until it reached zero strain. The electrical resistance also exhibited a delayed response to deformation, which may be attributed to the viscoelasticity of the PUR matrix, which is related to the duration of the time needed for the polymer chains to organize as opposed to the strain applied [66].

**Figure 7:**
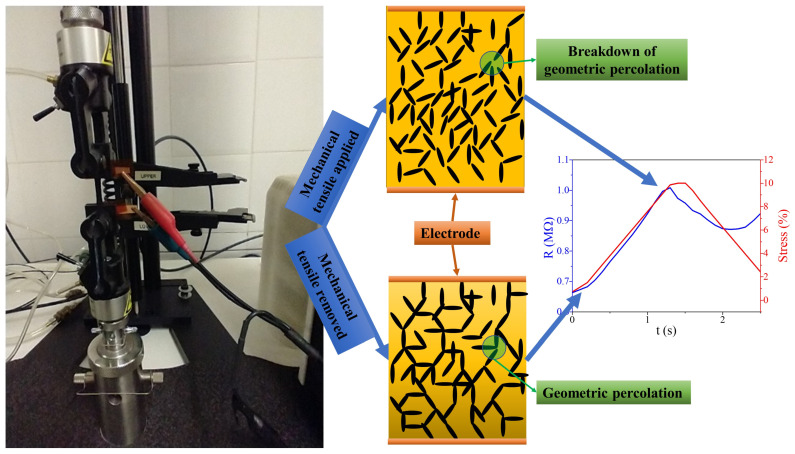
A schematic representation of the piezoresistivity test in conductive nanocomposites during stress cycles.

During all applied deformations, the piezoresistive behavior exhibited a good linearity between the electrical resistivity variation and strain cycles. As the deformation of the nanocomposite is increased by an external stimulus, the electrical resistance increases, while, as the deformation decreases, the electrical resistance decreases. Consequently, the initial increase in electrical resistivity of the PUR/MWCNT and PUR/CB nanocomposites samples can be attributed to the breakdown of the conductive percolation network formed by the MWCNT and CB, which leads to a decrease in the distribution of conduction paths. As the strain decreases (to zero) and the sample deformation returns to its initial length, the percolation paths are reconstructed and the electrical resistance decreases as well. However, because of the viscoelasticity of the PUR matrix, this decrease in resistance did not occur immediately upon removal of mechanical stress. For the different MWCNT and CB fractions, maximum strength was reached at different times under constant strain, as the alignment of MWCNT and CB particles occurred later for low concentrations of conductive fillers. Figure 7 illustrates the piezoresistivity test in conductive nanocomposites specimens during stress cycles, as well as the piezoresistive effect.

The same behavior was observed by Gonçalves et al. for a MWCNT/elastomer styrene-ethylene/butylene-styrene (SEBS) composite and by Costa et al. for polymer blends based on polyaniline (PANI) and SEBS [67,68]. Considering the outstanding piezoresistive properties observed by both groups during experimental testing, the samples have a high potential for advanced electromechanical sensor applications [67,68].

## 4. Conclusions

In this study, flexible films of PUR-based nanocomposites with nanoparticles of MWCNT and CB were obtained by the casting method and simple synthesis route. The SEM images showed that the MWCNT nanoparticles tended to agglomerate more than the CB nanoparticles, owing to van der Waals forces. This behavior corroborated the DC conductivity analysis, in which the PUR/CB nanocomposite exhibited a lower percolation threshold than that of the PUR/MWCNT nanocomposite (1.5 and 1.57, respectively). Both nanocomposites exhibited a nine-fold increase in *σ_dc_* compared to the PUR above the percolation threshold.

The *σ*′(*f*) spectra, as a function of the frequency, indicated that the PUR/MWCNT and PUR/CB nanocomposites with concentrations of the conductive phase above the percolation threshold had two distinct regions, with one independent and the other dependent on the frequency. The samples with concentrations below the percolation threshold exhibited a frequency-dependent behavior, which is characteristic of disordered systems in which charge carriers hop between localized states within conductive regions. Furthermore, the adjustment performed using the Jonscher’s equation for *σ*′(*f*) confirmed that the electric transport in the PUR/MWCNT and PUR/CB nanocomposites occurred via a hopping mechanism. The frequency-dependent behavior of $\varepsilon^{\prime }$ for PUR/MWCNT and PUR/CB could be attributed to the MWS polarization and space charge polarization in the bulk nanocomposite samples.

The PUR/MWCNT and PUR/CB nanocomposites with contents of 3, 4, and 5 mass% exhibited good piezoresistive properties up to a strain of 10%, which demonstrated their high stabilities and good electrical resistance responses. Thus, the PUR/MWCNT and PUR/CB nanocomposites have potential for applications as piezoresistive sensors.

## Figures and Tables

**Table 1:** Masses of MWCNT and CB to produce PUR/CB and PUR/MWCNT nanocomposites (in 3.0 g of PUR).

Samples	MWCNT (g)	CB (g)
PUR/MWCNT1	0.030	0
PUR/MWCNT2	0.061	0
PUR/MWCNT3	0.093	0
PUR/MWCNT4	0.125	0
PUR/MWCNT5	0.158	0
PUR/CB1	0	0.030
PUR/CB2	0	0.061
PUR/CB3	0	0.093
PUR/CB4	0	0.125
PUR/CB5	0	0.158

**Table 2:** Parameters obtained from fitting of the experimental data using the Jonscher’s equation for PUR/MWCNT and PUR/CB nanocomposite samples and neat PUR.

Samples	*σ_dc_* (S/m)	A	*n*	R^2^
**Neat PUR**	1.72 × 10^−11^	3.27 × 10^−11^	1.15	0.95
**PUR/CB (99/1)**	4.65 × 10^−10^	3.52 × 10^−11^	0.92	0.97
**PUR/CB (98/2)**	1.73 × 10^−7^	8.49 × 10^−10^	0.80	0.99
**PUR/CB (97/3)**	1.66 × 10^−6^	1.88 × 10^−9^	0.76	0.97
**PUR/CB (96/4)**	1.15 × 10^−4^	1.40 × 10^−8^	0.72	0.99
**PUR/CB (95/5)**	1.15 × 10^−3^	3.41 × 10^−7^	0.70	0.80
**PUR/MWCNT (99/1)**	7.01 × 10^−9^	5.38 × 10^−9^	0.86	0.96
**PUR/MWCNT (98/2)**	6.17 × 10^−4^	1.39 × 10^−8^	0.83	0.97
**PUR/MWCNT (97/3)**	6.65 × 10^−3^	4.06 × 10^−8^	0.79	0.95
**PUR/MWCNT (96/4)**	1.44 × 10^−2^	2.05 × 10^−8^	0.75	0.92
**PUR/MWCNT (95/5)**	1.21 × 10^−2^	1.08 × 10^−8^	077	0.90

**Table 3:** Dielectric parameters $\varepsilon^{\prime }$ and $\varepsilon^{\prime \prime }$ for conductive nanocomposites samples at frequencies of 1 kHz and 1 MHz.

Samples	* ε’ * (1 kHz)	* ε’ * (1 MHz)	*ε”* (1 kHz)	*ε”* (1 MHz)
PUR	3.52	6.23	0.82	39.2
PUR/MWCNT1	2.87	2.16	0.32	0.26
PUR/MWCNT2	4.72	2.21	1.15	0.41
PUR/MWCNT3	3.45	2.11	0.36	0.27
PUR/MWCNT4	11.01	5.30	1.88	0.86
PUR/MWCNT5	29.44	8.94	21.95	2.01
PUR/CB1	2.56	1.88	0.27	0.24
PUR/CB2	17.80	5.04	10.66	1.08
PUR/CB3	24.31	5.19	41.01	1.17
PUR/CB4	65.21	10.16	2801.92	8.64
PUR/CB5	70.14	20.12	20,515.62	31.32

## Data Availability

The data presented in this study are available upon request from the corresponding author.
